# Extracellular Vesicles Derived From Human Corneal Endothelial Cells Inhibit Proliferation of Human Corneal Endothelial Cells

**DOI:** 10.3389/fmed.2021.753555

**Published:** 2022-02-04

**Authors:** Mohit Parekh, Hefin Rhys, Tiago Ramos, Stefano Ferrari, Sajjad Ahmad

**Affiliations:** ^1^Institute of Ophthalmology, Faculty of Brain Sciences, University College London, London, United Kingdom; ^2^Flow Cytometry Science Technology Platform, Francis Crick Institute, London, United Kingdom; ^3^International Center for Ocular Physiopathology, Fondazione Banca Degli Occhi del Veneto, Venice, Italy; ^4^Cornea and External Eye Disease, Moorfields Eye Hospital NHS Foundation Trust, London, United Kingdom

**Keywords:** cornea, eye, exosomes, extracellular vesicles, corneal endothelial cells

## Abstract

Corneal endothelial cells (CEnCs) are a monolayer of hexagonal cells that are responsible for maintaining the function and transparency of the cornea. Damage or dysfunction of CEnCs could lead to blindness. Human CEnCs (HCEnCs) have shown limited proliferative capacity *in vivo* hence, their maintenance is crucial. Extracellular vesicles (EVs) are responsible for inter- and intra-cellular communication, proliferation, cell-differentiation, migration, and many other complex biological processes. Therefore, we investigated the effect of EVs (derived from human corneal endothelial cell line–HCEC-12) on corneal endothelial cells. HCEC-12 cells were starved with serum-depleted media for 72 h. The media was ultracentrifuged at 100,000xg to isolate the EVs. EV counting, characterization, internalization and localization were performed using NanoSight, flow cytometry, Dil labeling and confocal microscopy respectively. HCEC-12 and HCEnCs were cultured with media supplemented with EVs. Extracted EVs showed a homogeneous mixture of exosomes and microvesicles. Cells with EVs decreased the proliferation rate; increased apoptosis and cell size; showed poor wound healing response *in vitro* and on *ex vivo* human, porcine, and rabbit CECs. Thirteen miRNAs were found in the EV sample using next generation sequencing. We observed that increased cellular uptake of EVs by CECs limit the proliferative capacity of HCEnCs. These preliminary data may help in understanding the pathology of corneal endothelial dysfunction and provide further insights in the development of future therapeutic treatment options.

## Introduction

The cornea is the anterior tissue of the eye that refracts incident light to the lens, further converging it to the retina and optic nerve (1). Corneal clarity is essential for normal visual function. Corneal transparency is supported by its structural anatomy and physiology, mainly the endothelium. Human corneal endothelial cells (HCEnCs) line the under surface of the cornea. The cornea is avascular and receives its hydration and nutrients from the tear film and the aqueous humor from both sides of the eye. Excess accumulation of fluid, known as corneal oedema, affects corneal transparency and results in visual impairment. A mechanism to maintain corneal deturgescence is therefore required. This is performed by the corneal endothelium. The corneal endothelium acts as a barrier to fluid movement with the corneal endothelial cells actively pumping ions to move water osmotically from the aqueous humor to the corneal stroma and vice versa (2, 3). This combination of leaky barrier and fluid pump function is termed the pump-leak mechanism (2, 3).

HCEnCs have no mitotic activity *in vivo*, although they can be induced to divide in cultured corneal cells (4, 5). Human corneas at birth are characterized by a considerable endothelial cell reserve, with HCEnC density being 6,000 cells/mm^2^ at birth and declining to approximately 2,600 cells/mm^2^ or even low during the eighth decade of life (6, 7). HCEnCs have an almost perfect hexagonal shape which enables the formation of a tight cobblestone cell layer. The percentage of HCEnCs with a hexagonal shape also decreases from 75 to 60% with age (8). Other than age, there are several pathologies which result in accelerated HCEnC loss and dysfunction, resulting in loss of corneal clarity and blindness. These include viral infections, inflammation, and surgical procedures within the eye. However, dystrophies [Fuchs' dystrophy (9), posterior polymorphous dystrophy (10), congenital hereditary dystrophy (11)] or other conditions like iridocorneal endothelial syndrome (12) can also contribute toward partial or total blindness. Fuchs' endothelial corneal dystrophy (FECD) remains one of the common causes of corneal blindness resulting from the loss of endothelial cells. In FECD, the pump function of the endothelial cell decreases followed by a reduction in barrier function (9).

Several pathological processes require the migration and spreading of viable HCEnCs (13). In doing this, HCEnCs grow in size and lose their typical hexagonal shape. Endothelial wound healing is associated with a transient acquisition of fibroblast morphology, known as endothelial-mesenchymal transformation (14). In the later stages of endothelial healing, the number of tight junctions and pump sites return to physiological levels, corneal thickness as a result of corneal oedema returns to normal, and corneal transparency and vision is restored. When HCEnC density decreases below 500 cells/mm^2^, there is a significant risk of chronic and irreversible corneal oedema (15). In this scenario, the only treatment available is the replacement of the corneal endothelium by corneal transplantation (keratoplasty) from donor cadavers. Corneal endothelial failure remains one of the commonest reasons for requiring keratoplasty. Despite recent advances in surgical techniques, corneal transplantation has its limitations like transplant rejection and shortage of global donor cornea supply (16). Hence, HCEnC culture was introduced as a potential therapeutic option (17–19). However, HCEnCs are difficult to proliferate *in vitro* due to multiple factors like donor variability, cell source, age, preservation time (20), and tissue supply, which further challenges the conventional cell-based treatment option indicating the need of a parallel therapeutic approach.

Interestingly, while HCEnC's lack of proliferative capacity *in vivo* appears to be a feature found in humans, felines, and primates, many species such as rabbits and pigs have shown cell proliferation *in vivo*. Rabbits retain the ability to proliferate and regenerate *in vivo* following trauma (21–24). Pig corneal endothelial cells have also shown a higher proliferation potential compared to humans (25). Although pig corneal endothelium appears to have limited proliferative capacity *in vivo* compared to rabbits, their proliferative capacity remains better than HCEnCs (26). Hence, investigating the reasons for the proliferative capacity of these species and lack of proliferation in HCEnCs becomes important to develop new treatment options.

Extracellular vesicle (EV) trafficking is an important mechanism of intercellular communication in multicellular organisms (13–27). However, only a small collection of studies has examined EV function in the eye and the cornea in general (13, 28). Produced by different mechanisms with different subcellular origins and size distributions, three types of EVs have been classified: apoptotic bodies (1 to 5μm in diameter); microvesicles (up to 1μm in diameter); and exosomes (40 to 150 nm in diameter) (29). Exosomes are intraluminal membrane vesicles that form from the inward budding of the endosomal membrane. They contain different constituents of the parent cell, such as DNA, RNA, mRNA, micro-RNA, transcription factors, cytokines, lipids, metabolites, cytosolic and cell-surface proteins, and growth factors. Although the transfer of cargo between the cells is not completely understood, exosomes may be crucial in understanding cell trans-differentiation, proliferation, mechanisms or causes of disease, and to finding potential new therapies (29).

Due to the difficulties and limitations such as the global shortage of donor corneas, it is important to find alternative therapeutic strategies for treating corneal endothelial failure in humans. This requires basic understanding of the mechanisms that enable corneal endothelial maintenance in health and disease. As EVs have shown an important role in reprogramming normal/injured cells, the aim of this study was to investigate the role of EVs on HCEnCs and in other higher animals and identify the factors that inhibit the proliferation of these cells in humans *in vivo*.

## Materials and Methods

### Ethical Statement

Human donor corneas were shipped from Fondazione Banca degli Occhi del Veneto (FBOV, Venice, Italy) to UCL Institute of Ophthalmology (London, UK) with written consent for research use as the tissues were not suitable for transplantation due to poor endothelial cell count (<2200 cells/mm^2^). The tissues were utilized and discarded as per the Human Tissue Authority (HTA, UK) guidelines. The experiments were approved by the UCL ethics committee (10/H0106/57-2011ETR10) and were performed in accordance with the Declaration of Helsinki. The porcine and rabbit corneas were obtained from whole eyes shipped by a local abattoir and did not qualify for any special animal handling approval / ARVO guidelines for animal handling.

### HCEC-12 Cell Culture and Extraction of EVs

HCEC-12 cell lines were cultured on T-175 flasks (Nunc EasYFlask Delta surface, ThermoFisher Scientific, Waltham, MA, USA) using cell culture media (CCM-Ham's F12:Medium 199 (1:1) supplemented with 5% FBS; ThermoFisher Scientific, Waltham, MA, USA). Upon 95% confluence, the cells were starved with serum-depleted media (CCM without FBS; 10 mL per T-175 flask) every 24 h up to 72 h at 37°C, 5% CO_2_. Following starving, the conditioned media (CM) was collected every 24 h and centrifuged at 112 x g for 5 min at 4°C (centrifuge 5417, Eppendorf, Hamburg, Germany) to remove the dead cells and large debris. The supernatant was collected and re-centrifuged at 699xg for 10 min at 4°C to remove any potential media remnants. The CM was then filtered through a 0.22 μm filter (Merck Millipore, Burlington, Massachusetts, USA). Approximately 9 mL of the final volume from the flask was obtained. 4.5 mL of the filtered media was gently transferred to each sterile OptiSeal tube (Beckman Coulter, Brea, California, USA), capped and ultracentrifuged at 100,000xg in a TLA 100.4 fixed angle rotor (Beckman Coulter, Brea, California, USA) in an Optima Max-E ultracentrifuge machine (Beckman Coulter, Brea, California, USA) for 2 h at 4°C. The resulting pellet (volume dependent on experiment) was re-suspended and washed with sterile PBS followed by a second round of ultracentrifugation using the same settings as mentioned above, to obtain a pellet free of any media remnants. The resulting pellet was re-suspended in sterile PBS and either used directly for experiments or stored at −80°C. The entire procedure was carried out in the laminar flow hood to maintain sterility. The stored EV suspension was thawed in water bath at 37°C before use.

### Quantification, Characterization, Visualization, and Uptake of EVs

#### Quantification of EVs

From the EV suspension, 1 mL of the solution containing EVs was used for quantification and sizing following manufacturer's instructions (NanoSight NS300 instrument, Amesbury, UK). The temperature was kept constant at 22°C and the water viscosity kept at 0.953cP. For analysis, 1,498 frames were used at a rate of 25 frames per second. Sterile PBS was used as controls to ensure there was no contamination of any small visible molecules.

#### Characterization of EVs by Flow Cytometry

The EVs-containing suspension was ultra-centrifuged using the same settings as mentioned earlier and the resulting pellet was incubated with 10 μL of aldehyde/sulfate latex beads (ThermoFisher Scientific, Waltham, Massachusetts, USA) for 15 min at room temperature (RT). PBS was added to make up a final volume of 1 mL and the entire solution was incubated at 4°C overnight on a test tube rotator wheel fixed at 20 rpm (Stuart® Equipment, Saffordshire, ST15 OSA, UK). Glycine (Sigma-Aldrich, Darmstadt, Germany) was added to a final concentration of 100 mM and the resulting solution incubated at RT for 30 min. The solution was then centrifuged (Note: all centrifugation steps were performed for 3 min at 1800 x g in RT). The supernatant was removed, and the remaining pellet was washed thrice in 1 mL of 0.5% bovine serum albumin (BSA, Sigma-Aldrich, Darmstadt, Germany) in PBS. The pellet was re-suspended in 100 μL of primary antibody (Supplementary Table 1) diluted in 0.5% BSA and incubated in the dark for 30 min at 4°C. After washing and centrifugation, the resulting pellet was re-suspended in 100 μL of appropriate secondary antibody (Supplementary Table 2) diluted in 0.5% BSA. This suspension was then incubated in the dark for 30 min at 4°C. After washing and centrifugation steps, the resulting pellet was re-suspended in 500 μL of 0.5% BSA. This final suspension was analyzed using Fortessa X-20 (BD Biosciences, San Jose, CA, USA) flow cytometer (Laser 488 nm, filter 533/30) and the results were analyzed using BD FACSDiva software.

#### Cellular Uptake of EVs

The stored suspension of EVs was labeled with 1,1'-Dioctadecyl-3,3,3',3'-Tetramethylindocarbocyanine Perchlorate (DiI) fluorescent dye (V228885, ThermoFisher, Waltham, Massachusetts, USA). Briefly, the EV solution was gently mixed with Dil in PBS (1:1000) and incubated for 30 min in the dark at RT followed by a single wash with PBS and ultra-centrifugation (100,000xg) for 2 h at 4°C. The Dil-labeled EVs were diluted in the CCM supplemented with exosome depleted FBS (ThermoFisher Scientific) and used for qualitative and time point analysis.

For confocal microscopy, approximately 50,000 cells (HCEC-12) per well of 4-well lab-Tek II chamber slides (Thermo Fisher Scientific) and for Imagestream flow cytometry analysis, approximately 150,000 cells per well of a 12 well plate (Thermo Fisher Scientific) was cultured for 48 h. HCEC-12 cells were refreshed with the CM (fetal bovine serum (FBS) replaced with exosome-free serum) supplemented with 40 μL of EVs (obtained from 1 mL of the EV suspension i.e., ~5 X 10^6^ particles) with 360 μL of CM (10% EVs). The cells were monitored at different time points i.e., 3, 6, 12, 24, and 48 h. The media was not refreshed after adding the EVs throughout the entire course of this experiment. Negative control was cells with standard FBS.

#### Cellular Uptake and Localization of EVs-Time Point Analysis Using Confocal Microscope

The cells (control and with EVs) were washed with PBS and fixed with 4% paraformaldehyde (PFA) at 3, 6, 12, 24, and 48 h following addition of Dil-positive EVs. Hoechst 33342 (ThermoFisher Scientific) (0.5 μg/mL) was added on the cells to stain the nucleus at RT for 30 min. After each step, the cells were washed at least twice with PBS. After detaching the walls of the Lab-Tek slides, the cells were covered with mounting media (Vectashield, Vector Laboratories, Burlingame, CA, USA) and cover slips. The cells with EV uptake were imaged using the LSM 700 confocal microscope (Carl Zeiss, Cambridge, UK) and captured using a built-in Zen software. Localization was observed using 3D view feature of the confocal microscope following z-stacking of the image.

#### Internalization and Cellular Uptake of EVs Using Imagestream

The cells (control and with EVs) were washed with PBS and detached from the plate using TrypLE Express (1X), phenol red (Life Technologies, Monza, Italy) treatment for 5 min at 37°C to dissociate the clumps into single cells. The collected cells were centrifuged at 194xg for 5 min, washed and fixed with 4% PFA. The fixed cells were labeled with Hoechst 33342 (as described above) in 1.5 mL Eppendorf tubes, washed with PBS, and re-suspended in 50 μL of PBS. Samples were acquired on an ImageStream^x^ MkII (Austin, Texas, USA) at 60x magnification on low flow rate. The 405, 561, and 785 nm (for scatter) lasers were switched on and set to 30, 200, and 1.0-1.2 mW, respectively. Laser powers were chosen that maximized resolution while avoiding pixel saturation. Channels 1 and 9 were reserved for brightfield images. Using the IDEAS analysis software, single cells were gated using Area vs. Aspect ratio (a measure of object roundness). The gradient RMS feature of the brightfield images was used to gate on focused events. Percentage of Dil+ events were identified from the different phases after gating on non-clipped objects using the Raw Centroid X and Hoechst 33342-positive events. The quantification of internalization, total EV+ population and total internalization score was obtained from these positive events. The internalization feature is defined as the ratio of the intensity inside the cell to the intensity of the entire cell. The higher the score, the greater the concentration of intensity inside the cell. All pixels were background-subtracted and an Adaptive Erode (M01, Ch01 BF1, 78) mask was created to define the inside of the cell for this feature. The Bright Detail Intensity R3 and Bright Detail Intensity R7 features computed the intensity of localized bright spots within the masked area in the image. Bright Detail Intensity R3 and R7 features compute the intensity of bright spots that are 3 pixels or 7 pixels in radius or less, respectively. In each case, the local background around the spots was removed before the intensity computation.

### Human Corneal Endothelial Cell Line (HCEC-12) Culture With EVs

Human corneal endothelial cells from a certified cell line (HCEC-12) were cultured on 75 cm^2^ culture flasks (Nunc, Thermo Fisher Scientific, Rochester, NY, USA) to reach 95% confluence using CCM as mentioned above. The cells were trypsinised and cultured on Lab-Tek II chamber slides (8 chambers, 25 x 75 mm, 0.7 cm^2^ culture area, Thermo Fisher Scientific). Upon confluence, the CCM was removed and the HCEC-12 cells were washed with sterile PBS. The cells were refreshed with CCM (as control) and media supplemented with 10% EVs, as described above (CCM with exo-free serum-as experimental group). The cells were analyzed for proliferation rate, doubling time, viability, apoptosis and endothelial cell specific markers at different time points.

### Human Corneal Endothelial Cell Culture From Old-Aged Donor Tissues With EVs

#### Endothelial Cell Evaluation

Donor endothelium of all the tissues was stained with trypan blue (0.25% w/v) to determine the viability of the cells. Approximately 100 μL of trypan blue was applied topically on the endothelial surface for 20 s and washed with sterile phosphate buffered saline (PBS). The endothelium was exposed to a hypotonic sucrose solution (1.8%) to count the number of endothelial cells using a reticule (10 x 10) fixed to the eyepiece of an inverted microscope (Nikon Eclipse TS100, Nikon, Surrey, UK). An average of five different counts was recorded (30).

#### Cell Culture

The Descemet's membrane-endothelial complex of the tissues were stripped in multiple pieces to ensure quick enzymatic digestion. The excised pieces were digested in 2 mg/mL collagenase Type 1 (Thermo Fisher Scientific, Rochester, NY, USA) for 2 h at 37°C and 5% CO_2_. The resulting solution was centrifuged for 5 min at 194xg and the pellet was re-suspended with TrypLE Express (1X), phenol red (Life Technologies, Monza, Italy) for 5 min at 37°C to further dissociate into single cells. The supernatant was discarded, and the cells were re-suspended in 200 μL of the HCEnC culture medium (HCM), which is a formulation of 1:1 Ham's F12:M199 (Sigma-Aldrich), 5% FBS, 20 μg/ml ascorbic acid (Sigma-Aldrich), 1% Insulin Transferrin Selenium (Gibco), 10 ng/ml recombinant human FGF basic (Gibco), 10 μM ROCK inhibitor (Y-27632; Miltenyi Biotech) and 1% PenStrep (Sigma-Aldrich) (18, 31–35). The cells were counted using haemocytometer. Lab-Tek II chamber slides (8-well) were coated with 50 μL Fibronectin Collagen (FNC) coating mix (US Biological Life Sciences, Salem, Massachusetts, USA) for 30–45 min at 37°C and 5% CO_2_. The residual coating was removed before plating cells. 200 μL of the cell suspension from each cornea was divided into two equal halves and plated on each chamber a) without EVs and b) with EVs (10%). The media was topped up to make a final volume of 400 μL. The HCM (with/without EVs) was replaced, and the cells were monitored every alternate day until confluence followed by end-stage characterization.

### Proliferation Rate, Cell Doubling Numbers and Time on HCEC-12 and HCEnCs

The proliferation rate was measured every alternate day using an in-built reticule (10 x 10) attached to an inverted microscope (Nikon Eclipse TS100; Nikon). The number of endothelial cells/mm^2^ were counted using the same reticule determined by counting the number of blocks filled by the cells every alternate day represented as percentage of proliferation rate in the given area. This also facilitated in calculating the cell doubling time and doubling numbers.

### Hoechst 33342, Ethidium Homodimer and Calcein AM (HEC) Staining to Determine Live/Dead HCEC-12 Cells and HCEnCs

Cells at confluence were washed with PBS after preservation prior to the assay. 5 μL of Hoechst 33342 (H) (Thermo Fisher Scientific), 4 μL of Ethidium Homodimer EthD-1 (E) and 2 μL Calcein AM (C) (Live/Dead viability/cytotoxicity kit, Thermo Fisher Scientific) was mixed in 1 mL of PBS. 100 μL of the final solution was directly added on the cells and incubated at room temperature in dark for 45 min, followed by a single washing step with PBS. The walls of the Lab-Tek slides were detached and the cells were mounted with mounting media (without DAPI). The Zeiss LSM 700 confocal microscope (Carl Zeiss, Cambridge, UK) was used to image the cells that were captured using built-in Zen software. The measurements and data analysis were performed using ImageJ (FIJI) bundled with 64-bit Java 1.8.0 112. Viability of cells was measured as the number of Calcein AM-Hoechst-positive cells (double stained) compared with the number of only Hoechst-positive cells. The images were split and the Hoechst positive cells were overlayed with numbers. Calcein AM positive cells were patched on the overlayed image to calculate the number of cells with no calcein positivity and converted to percentage for statistical analysis.

### Cell Apoptosis Using Terminal Deoxynucleotidyl Transferase Deoxyuridine Triphosphate Nick-End Labeling Assay on HCEC-12 and HCEnCs

Cell apoptosis was performed as described in the manufacturer's protocol for TACS 2 terminal deoxynucleotidyl transferase (TdT) diaminobenzidine (DAB) *in situ* apoptosis detection kit (Cat# 4810-30-K; Trevigen, Maryland, USA). One separate positive sample was induced with apoptosis using TACS nuclease and all the samples were viewed and imaged using inverted light microscope (Nikon Eclipse TS100, Nikon, Surrey, UK). The apoptotic cells were manually counted, and an average was recorded from five random areas (36).

### Immunostaining of Zonula Occludens-1 (ZO-1) and Na^+^/K^+^ATPase

Cells at confluence were washed with PBS and fixed in 4% paraformaldehyde (PFA) at RT for 20 min. The cells were permeabilized with 0.25% Triton X-100 in PBS for 30 min. After blocking with 10% goat serum for 1 h at RT, the cells were incubated overnight at 4°C with primary antibody anti-ZO-1 (ZO-1-1A12, Alexa Fluor 488; Thermo Fisher Scientific, Rochester, NY, USA) (HCEC-12 and HCEnCs), 1:200 and; anti-Na/K ATPase (Sodium Potassium ATPase Alpha 1 Antibody (464.6)–FITC; Novus Biologicals, Centennial, CO) (HCEnCs only), 1:50. Hoechst 33342 (0.5 μg/ml) was diluted in PBS and 100 μL of the solution was added on the cells to stain the nucleus. After each step, the cells were washed 3 times with PBS. After detaching the walls of the Lab-Tek slides, the cells were covered with mounting medium and cover slips. Expression of these markers were examined using the LSM 700 confocal microscope (Carl Zeiss) and images were captured using an in-built Zen software.

For hexagonality, ZO-1-positive images were converted to overlay masks using pre-determined macroinstructions to define the parameters of both hexagonality and polymorphism within a particular area (37). The images were auto-converted and the total number of cells in the investigated area were counted using the macros for ZO-1. The hexagonal and polymorphic cells were counted manually depending on the cellular structure comprising 6 borders per cell for hexagonal cells and < 4 borders for severely polymorphic cells in the investigated area. Cell area (μm^2^) was measured by marking the borders of the cell using a free-hand tool followed by the area measurement tool. The numbers were converted into percentage for statistical analysis.

### Effect of EVs on Wound Healing (Scratch Assay)–*in vitro* and *ex vivo* (Human, Porcine and Rabbit Corneal Tissues)

#### *In vitro* Wound Healing of HCEC-12 Cells

HCEC-12 cells were cultured on standard 12 well plates. Upon confluence, the center of the wells was scratched using a 1 mL pipette tip to create a wound. The well was washed using sterile PBS and the cells were refreshed with CCM supplemented with EVs (10% EVs), as described above. The cells in exo-free serum CCM were considered as control. The wounded area was monitored every 24 h till the wound healing was complete. The images were loaded on ImageJ and the total area of the wounded zone was measured at different time point leading to calculation of percentage wound closure at each time point.

#### *Ex vivo* Wound Healing on Human Donor Cornea

Like the *in vitro* wound healing assay, a scratch was made at the center of the tissue using a 1 mL pipette tip. The tissue was washed and placed in HCM supplemented with/without EVs. The tissues with exo-free HCM were considered as control. The wound healing was monitored every 24 h and calculated as mentioned above.

#### *Ex vivo* Wound Healing on Porcine and Rabbit Corneas

Porcine and rabbit eyes were obtained from a local abattoir. The corneas were excised and preserved in CCM before the experiments. Using the same technique as described above, the corneal endothelium was scratched at the center. The tissues (donor-matched i.e., OD vs. OS) were washed and preserved in the media supplemented with exo-free CCM and the other tissues were preserved with 10% HCEC-12 derived EVs. The wound healing was monitored every 24 h and calculated as mentioned above.

### Cargo Characterization by Next Generation Sequencing

One μL of total EV-RNA was utilized for measurement of small RNA concentration by Agilent Bioanalyzer Small RNA Assay using Bioanalyzer 2100 Expert instrument (Agilent Technologies, Santa Clara, CA). Next generation sequencing libraries were generated with the TailorMix Micro RNA Sample Preparation version 2 protocol (SeqMatic LLC, Fremont, CA). Briefly, 3'-adapter was ligated to the RNA sample and excess 3'-adapters were removed subsequently. 5'-adapter was then ligated to the 3'-adapter ligated samples, followed by first strand cDNA synthesis. cDNA library was amplified and barcoded via enrichment PCR. Final RNA library was size-selected on an 8% TBE polyacrylamide gel. Sequencing was performed on the Illumina NextSeq 500 platform at a read length of 1 x 75 bp single-end at SR50. FASTQ files for each sample were generated using bcl2fastq software (Illumina Inc., San Diego, CA). FASTQ data were checked using FastQC tool and Bowtie2 used to map the spike-in DNA. RNA adapters were trimmed off using FastqMcf and cutadapt, with PRINSEQ used in the quality filtering step. Bowtie was used to map against the human reference genome (GRCh37). DEseq was used for abundance determination and differential expression analysis (38–41). The miRNA database was added, and the pathway analysis was performed using TarBase v7.0 of KEGG analysis (mirPath v.2, Diana tools). The heatmap was created after adding all the miRNAs in the KEGG analysis and selecting pathways union with settings of *p*-Value threshold at 0.05 using enrichment analysis method of Fisher's Exact Test (Hypergeometric Distribution).

### Statistical Analysis

A two-tailed Wilcoxon signed rank test for paired test and Mann-Whitney test was used to evaluate the evidence of a difference between the cells *in vitro* and *ex vivo* with and without EVs. All statistical analyses were conducted using GraphPad Prism 5.01 software.

## Results

### Data From Human Donor Corneal Endothelial Cells (*n* = 40)

Average age of 62.75 ± 6.22 (mean ± SD) years, endothelial cell density of 1,800 ± 95.34 cells/mm^2^, post-mortem interval of 12.48 ± 5.68 h with preservation time of 12.08 ± 4.76 days from the donor corneas was recorded.

### Quantification and Characterization of EVs From HCEC-12 Cells

#### NanoSight Analysis (n = 3)

**Figure 1:**
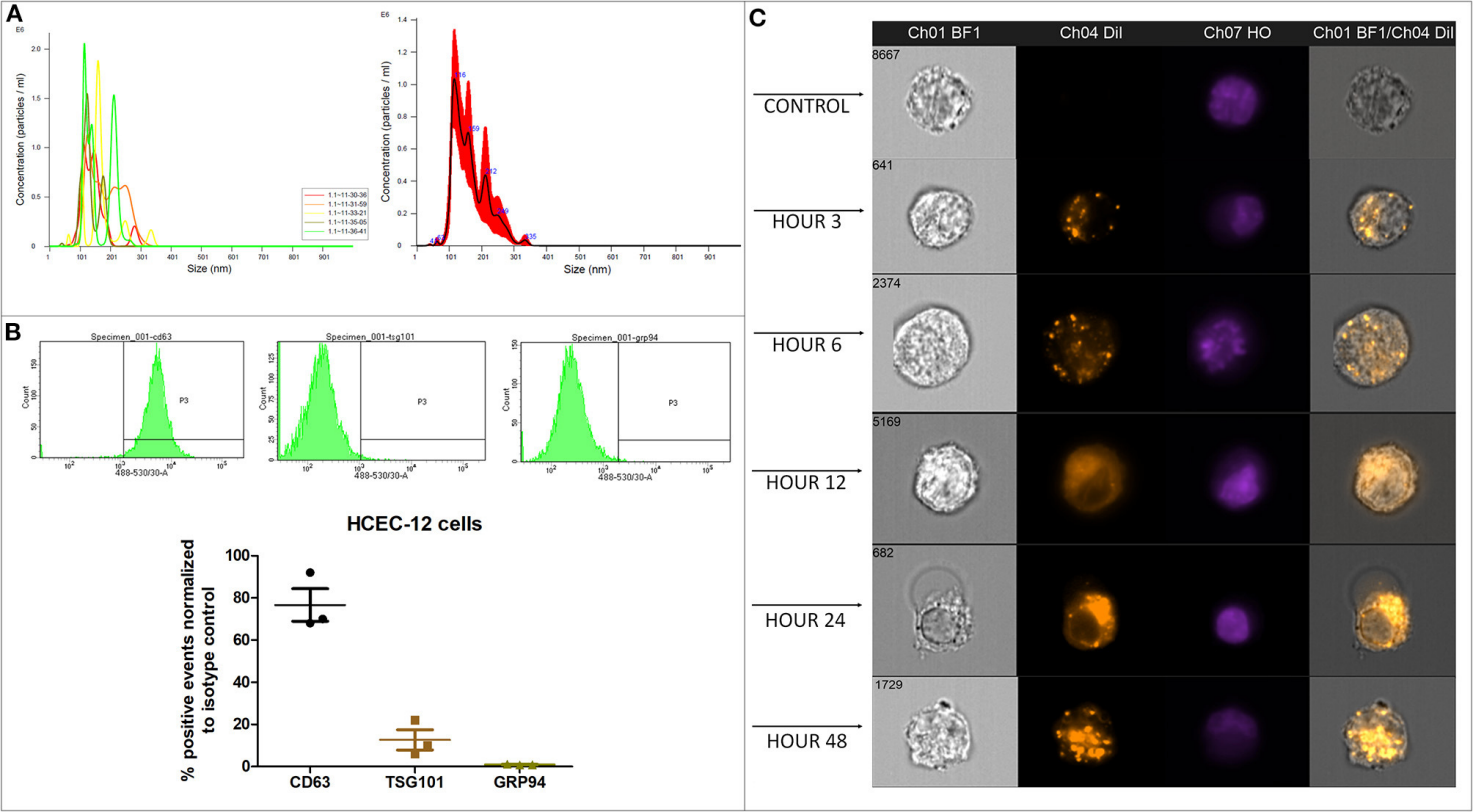
Quantification, characterization and, internalization of EVs. **(A)** Representative Nanosight analysis of the EVs derived from HCEC-12 cell lines. The peak is in the acceptable exosomal size range however, there are likely other particles such as microvesicles present in the sample. Apoptotic bodies seem unlikely as all the particles are <400 nm. **(B)** Flow cytometry analysis showing CD63 and TSG101 positivity and negative expression of GRP94. These markers and expression indicate that the EV samples have a heterogeneous population including exosomes. **(C)** Imagestream analysis showing cellular uptake and internalization of Dil labeled EVs at different time points.

Average concentration of 1.23 x 10^9^ ± 5.63 x 10^7^ particles/mL were found from approximately 18 million cells using Nanosight analysis i.e., ~68 EVs per cell. The particles were distributed in the size range of exosomes and microvesicles (Figure 1A, Supplementary Video 1).

#### Flow Cytometery (n = 3)

Flow cytometry analysis showed that the EV samples were positive for CD63 and TSG101 and did not express GRP94 (Figure 1B).

Quantification and characterization indicated that the extracted EV solution contained a heterogenous population of exosomes and microvesicles.

### Cellular Uptake and Internalization of EVs Using Dil Labeling and Imagestream Analysis on HCEC-12 Cells

#### Confocal Imaging (n = 3)

Dil labeled the lipid bilayer of the EVs. The 3D image showed that the EVs were internalized inside the cell either on or surrounding the nucleus or in the cytoplasmic region within 24 h (Supplementary Figure 1A). The EVs were visible inside the cells as early as 3 h after addition (Supplementary Figure 1B). A gradual increase in the uptake of EVs was observed between 3 and 48 h (Supplementary Figure 1B).

#### Imagestream Analysis (n = 3)

Dil-labeled EVs showed internalization by 3 h. However, Dil uptake and internalization of EVs was at its peak at 48 h (Figure 1C, Supplementary Figure 1C). The localization was not specific to a particular cellular organelle and was distributed throughout the cell.

### Effect of EV Uptake on Proliferation, Cell Numbers and Doubling Time of HCEC-12 Cells and HCEnCs

#### HCEC-12 (n = 40)

**Figure 2:**
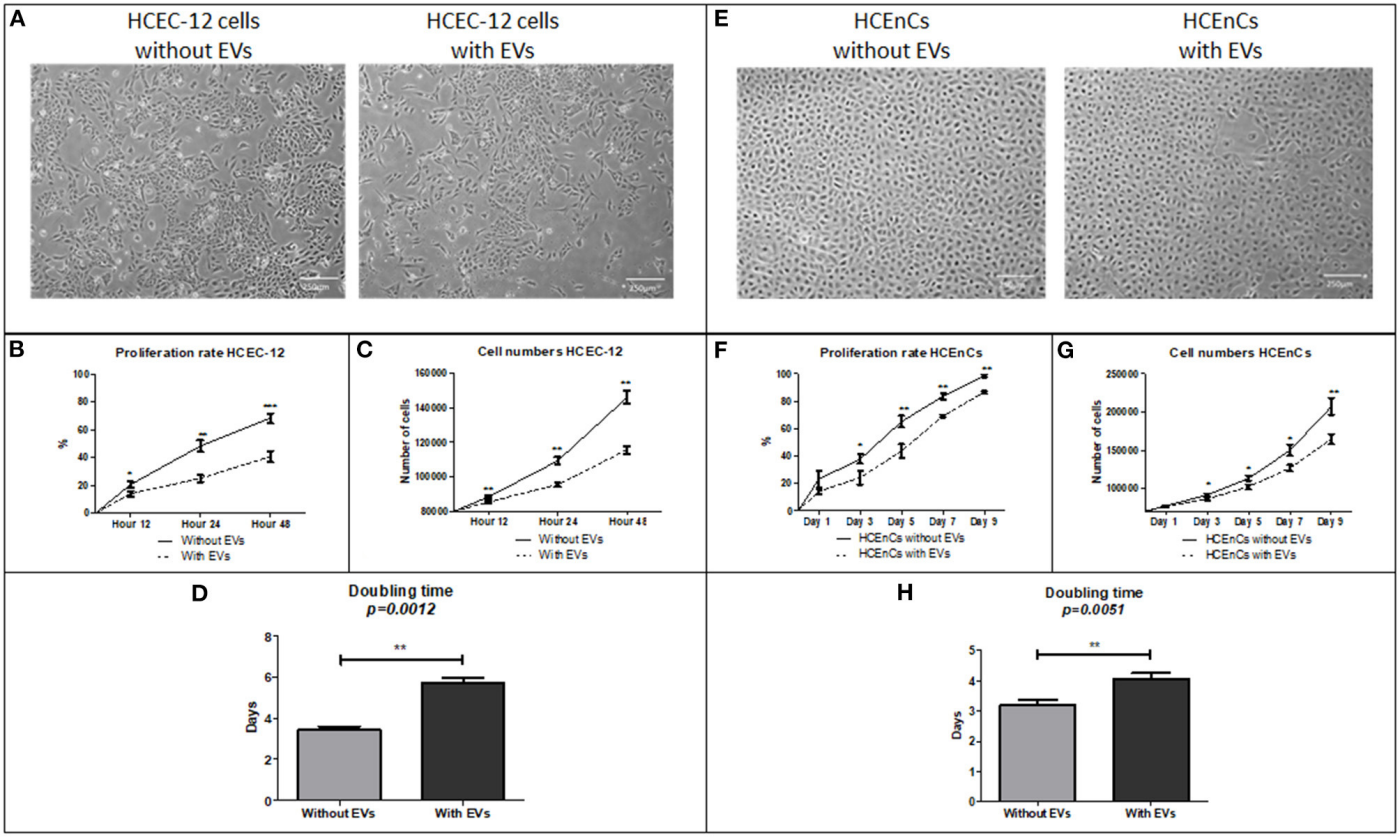
Morphology, proliferation rate, cell doubling time and rate on HCEC-12 and HCEnCs. **(A)** Morphology of cells was found to be normal without much changes in cellular shape or size. This also helped in checking the confluence rate of cells. **(B)** Statistically significantly higher proliferation rate was observed in cells without EVs. **(C)** Cell numbers significantly increased and **(D)** cell doubling time significantly decreased in the absence of EVs. **(E)** morphological difference was observed in cells with EVs. However, the cells were fully confluent by day 9 without EVs. **(F)** Statistically significantly higher proliferation rate was observed in cells without EVs. **(G)** Cell numbers significantly increased and **(H)** cell doubling time significantly decreased in the absence of EVs. Scale = 250 μm. *p < 0.05; **p < 0.01; ***p < 0.001.

Proliferation rate of HCEC-12 cells without EVs was significantly higher compared to the cells with EVs at 12, 24, and 48 h (Figures 2A,B). The cell numbers significantly increased from 80,000 cells to 145,000 in cells without EVs compared to 115,000 in cells with EVs (Figure 2C). Cell doubling time from the cells without EVs group was significantly less i.e., <4 days compared with the EVs group, which was over 6 days (Figure 2D) (Table 1).

**Table 1:** Analysis of parameters such as proliferation rate, doubling time and rate, live/dead and apoptosis, hexagonality and cell area.

**HCEC-12**	**Without EVs**	**With EVs**	**HCEnCs**	**Without EVs**	**With EVs**
**Proliferation (%)**			**Proliferation (%)**		
Hour 12	21 ± 2	14 ± 3	Day 1	23 ± 6	14 ± 2
Hour 24	48 ± 4	25 ± 3	Day 3	38 ± 3	24 ± 5
Hour 48	68 ± 3	40 ± 4	Day 5	65 ± 4	44 ± 6
			Day 7	84 ± 2	69 ± 2
**Cell doubling (no. of cells)**			Day 9	98 ± 2	87 ± 1
Hour 12	88,400 ± 690	85,333 ± 832			
Hour 24	109,180 ± 2,106	95,433 ± 1,322	**Cell doubling (no. of cells)**		
Hour 48	145,760 ± 3,678	115,615 ± 2,196	Day 1	77,250 ± 760	75,800 ± 483
			Day 3	91,370 ± 1,655	86,116 ± 1,794
Doubling time (days)	3 ± 1	6 ± 1	Day 5	113010 ± 3214	101,910 ± 2,854
			Day 7	149,832 ± 7,238	126,516 ± 4,190
Live (%)	96 ± 1	94 ± 2	Day 9	207,050 ± 11,010	164,616 ± 6,294
Dead (%)	1 ± 1	2 ± 1			
Apoptotic (%)	2 ± 1	3 ± 1	Doubling time (days)	3 ± 2	4 ± 1
Hexagonality (%)	72 ± 6	69 ± 7	Live (%)	95 ± 1	92 ± 2
Cell area (%)	407 ± 18	427 ± 16	Apoptotic (%)	4 ± 2	8 ± 3
			Hexagonality (%)	72 ± 3	67 ± 5
			Cell area (%)	401 ± 25	443 ± 33

#### HCEnCs (n = 40)

HCEnCs without EVs showed 99% confluency by day 9 compared to 88% confluence observed in cells with EVs (Figure 2E). Proliferation rate was significantly higher in cells without EVs compared to the cells with EVs at days 3, 5, 7 and 9 (Figure 2F). The cell numbers significantly increased from 70,000 cells to 200,000 in cells without EVs compared to 160,000 in cells with EVs (Figure 2G). Cell doubling time from the cells without EVs was significantly less i.e., <4 days compared with the EVs group, which was over 4 days (Figure 2H) (Table 1).

Corneal endothelial cells with EVs inhibited the proliferation of cells and increased the cell doubling time.

### Effect of EV Uptake on Viability of Cells Using HEC Staining

#### HCEC-12 (n = 6)

**Figure 3:**
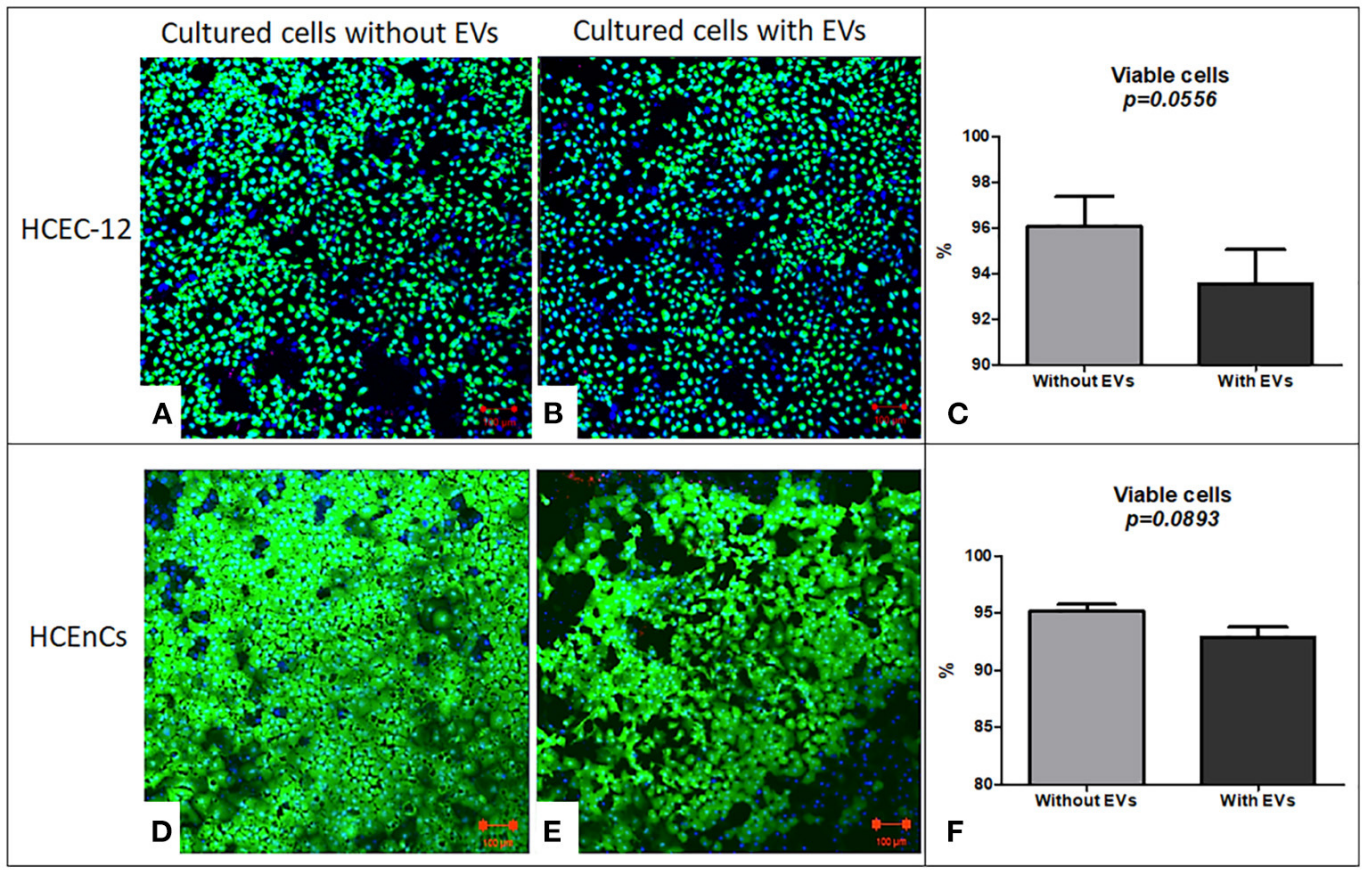
Live/dead analysis using Hoechst, Ethidium homodimer and Calcein AM staining (HEC) / triple labeling. In HCEC-12 lines **(A)** higher number of viable cells were observed in cells without EVs compared with that **(B)** with EVs. **(C)** The percentage viability was not found to be significantly different in cells with or without EVs. In HCEnCs, a similar trend was observed i.e., a higher number of viable cells **(D)** without EVs compared to **(E)** with EVs showing no statistical significance in **(F)** viability. Ethidium homodimer positive cells were not observed. (Hoechst, nuclear in blue staining and Calcein AM, live cells in green staining). Scale = 100 μm.

HEC staining showed viability (calcein AM positive cells) in both, cells without (Figure 3A) and with EVs (Figure 3B). The cells without EVs showed a higher number of viable cells compared to the cells with EVs (Figure 3C), although it was found to be non-significant.

#### HCEnCs (n = 6)

HEC staining showed viable cells (calcein am positive cells) in both, cells without (Figure 3D) and with EVs (Figure 3E). The cells without EVs showed a higher number of viable cells compared to the cells with EVs (Figure 3F), but not found to be significantly different.

This indicated that addition of 10% EVs in the cells does not affect the cell viability (Table 1).

### Effect of EVs on Cell Apoptosis Using TUNEL Assay

#### HCEC-12 (n = 6)

**Figure 4:**
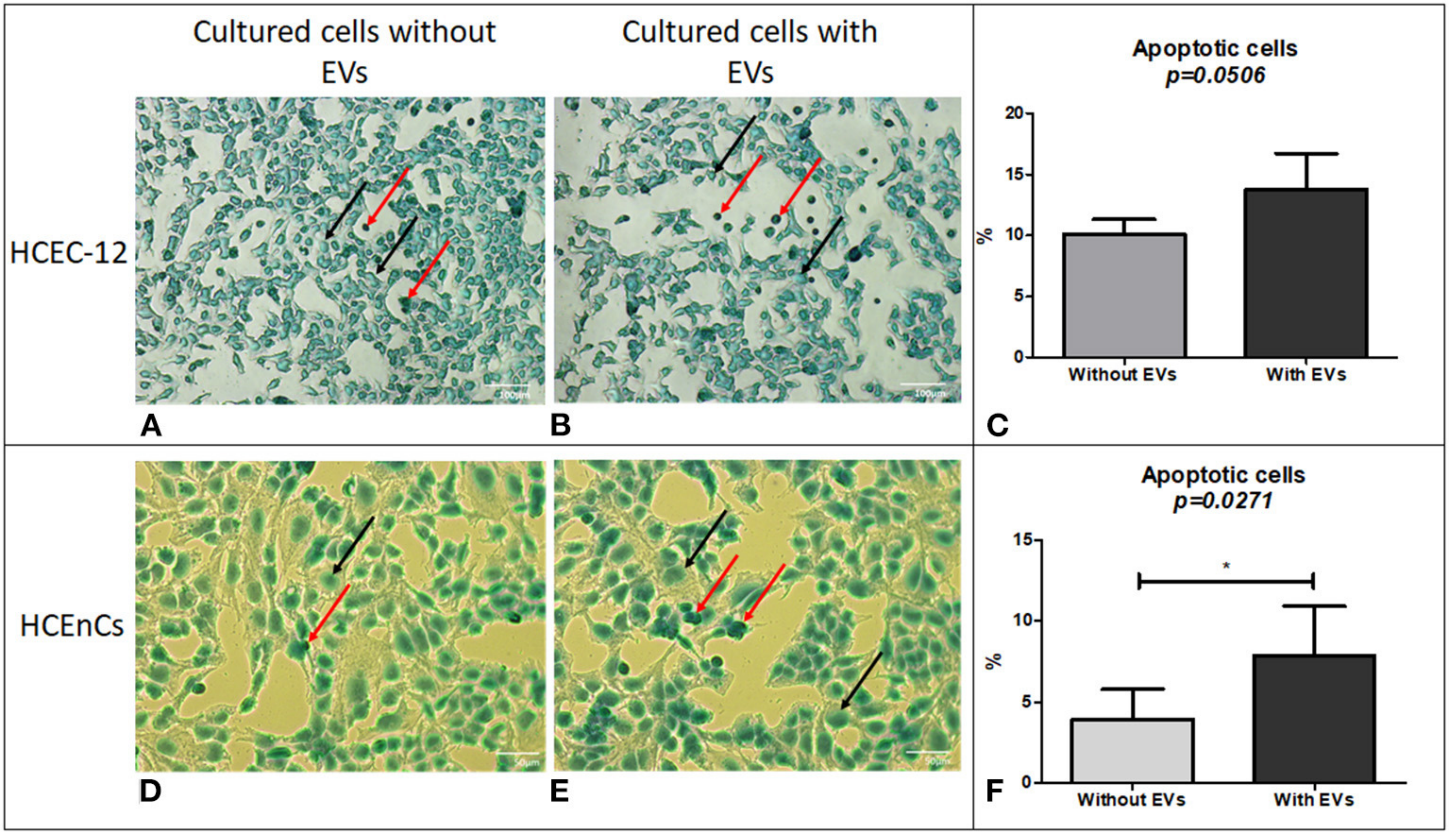
Cell apoptosis using TUNEL assay. Although a lower number of cells were found to be apoptotic in HCEC-12 **(A)** without EVs compared **(B)** with EVs, **(C)** a statistical significance was not observed between the two groups. However, HCEnCs showed lower number of apoptotic cells **(D)** without EVs compared to **(E)** with EVs and was found to be **(F)** significantly different. (Black arrow, Methyl green counterstain; red arrow, apoptotic cells). Scale: **(A,B)** = 100 μm; **(D,E)** = 50 μm. *p < 0.05.

Cells without EVs (Figure 4A) and with EVs (Figure 4B) showed apoptotic cells. However, they were not found to be statistically significantly different (Figure 4C) (Table 1).

#### HCEnCs (n = 6)

Cells from human donor tissues cultured without EVs (Figure 4D) showed a significantly lower number of apoptotic cells compared with the cells containing EVs (Figures 4E,F).

The data indicated that EVs could contain pro-apoptotic factors that induce apoptosis in human donor corneal endothelial cells.

### Effect of EVs on Expression of Specific Proteins-ZO-1 Staining for Tight Junctions and Analysis of Hexagonality and Cell Area, and Na^+^/K^+^-ATPase for Pump Functions

#### ZO-1 Staining on HCEC-12 (n = 6)

**Figure 5:**
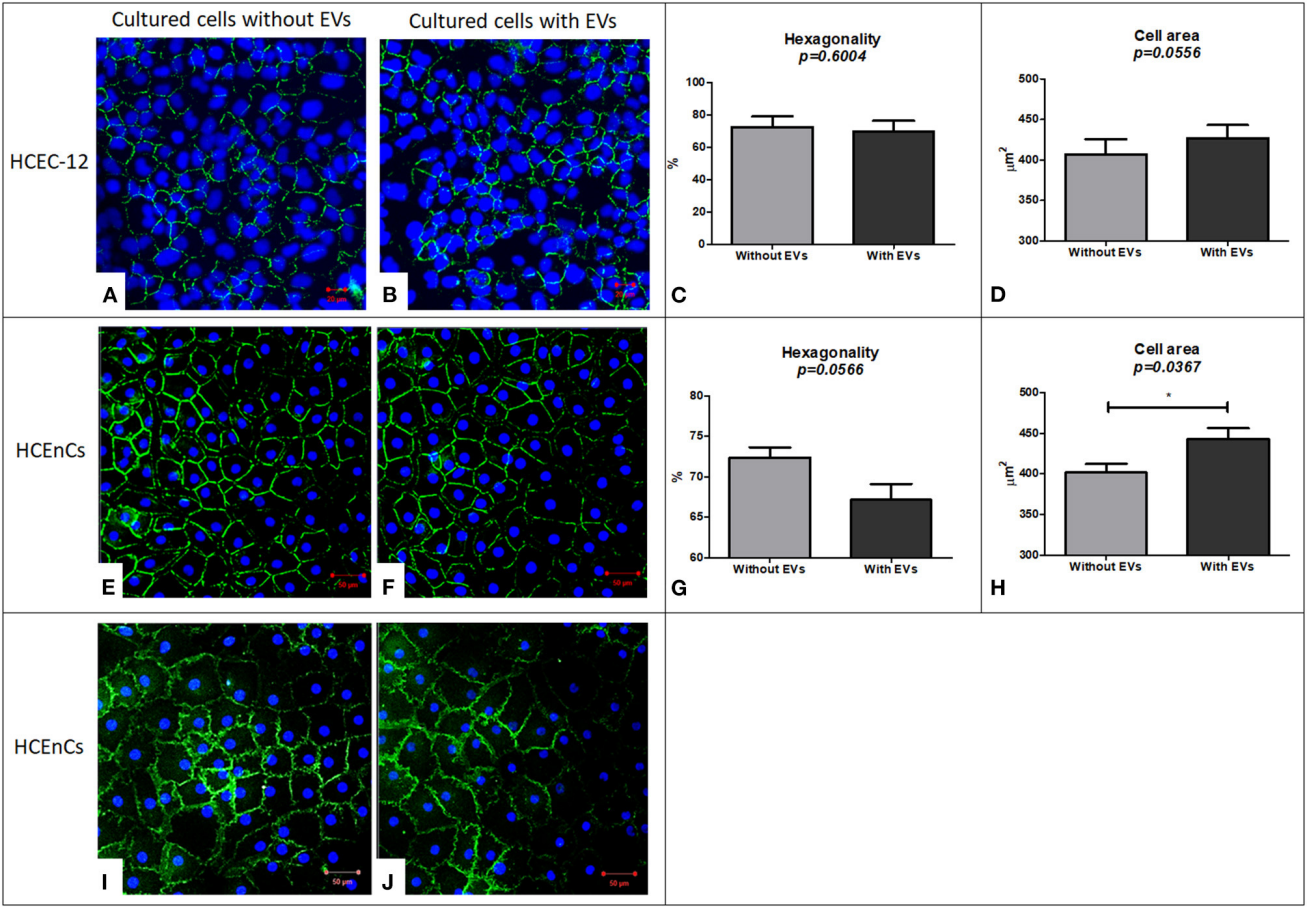
Immunostaining using ZO-1 biomarker for expression and evaluation of hexagonality and cell area and expression of Na^+^/K^+^-ATPase biomarker to study the functionality of the cells. HCEC-12 cells showed expression of ZO-1 marker in both the cells. **(A)** without EVs and **(B)** with EVs with no significant difference found in terms of **(C)** hexagonality or **(D)** cell area. Similarly, ZO-1 was expressed in HCEnCs **(E)** without EVs and **(F)** with EVs. Although the cells did not show a significant difference in terms of **(G)** hexagonality, it was found to be significantly different in terms of **(H)** cell area. The cells **(I)** without EVs and **(J)** with EVs showed the expression of pump functions in terms of positive expression of Na^+^/K^+^-ATPase marker. Scale: **(A,B)** = 20 μm; **(E,F)** and **(I,J)** = 50 μm. * *p* < 0.05.

HCEC-12 cells without EVs (Figure 5A) and with EVs (Figure 5B) showed the expression of ZO-1. As corneal endothelial cells are a monolayer of hexagonal cells, it is important to determine the hexagonality of these cells, which further indicates whether the cells are differentiating into other cell types or maintaining their phenotype after addition of EVs. There was no significant difference between the cells with and without EVs in terms of hexagonality (Figure 5C) or cell area (Figure 5D) (Table 1). ZO-1 expression was lost at multiple sites in both groups.

#### ZO-1 Staining on HCEnCs (n = 6)

HCEnCs without EVs (Figure 5E) and with EVs (Figure 5F) expressed ZO-1. Although there was no significant difference between the cells with and without EVs in terms of hexagonality (Figure 5G), the cells without EVs showed significantly smaller cell area compared to the cells with EVs (Figure 5H) (Table 1). ZO-1 expression was found to be homogeneously distributed at the intercellular junctions in the sample without EVs compared to loss of ZO-1 expression at multiple sites with EVs indicating that the EVs may influence the development and maintenance of tight junctions. However, this must be further investigated as it is a subjective evaluation.

#### Na^+^/K^+^-ATPase Staining on HCEnCs (n = 6)

As a functional marker, Na^+^/K^+^-ATPase was expressed in cells without EVs (Figure 5I) and with EVs (Figure 5J). However, it was not expressed throughout the sample in the presence of EVs indicating that EVs may also influence the pump-function outcomes of HCEnCs. This staining was not performed on cell lines as it was only used to determine the effect of EVs on the function of HCEnCs.

### Effect of EVs on Corneal Endothelial Wound Healing

#### Wound Healing Rate on HCEC-12 Cells (n = 6)

**Figure 6:**
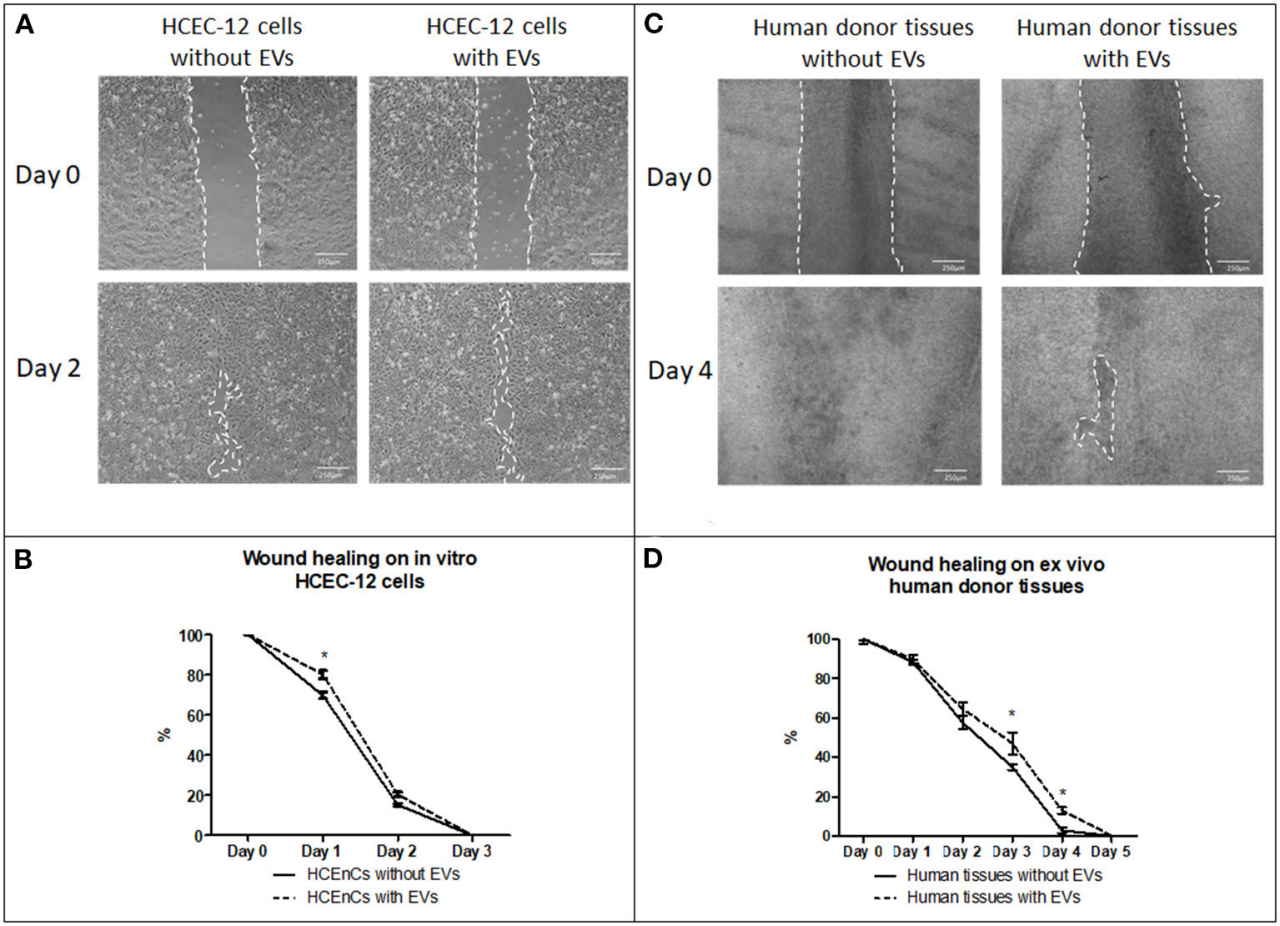
Effect of EVs derived from HCEC-12 cells on *in vitro* cells and *ex vivo* human donor corneas –scratch assay. **(A)** wound healing on HCEC-12 cell line showing slow response in presence of EVs. **(B)** data showing early slow wound healing response on HCEC-12 cells in presence of EVs. **(C)** Wound healing response on *ex vivo* human donor tissues without and with EVs. **(D)** data showing statistically significantly slow wound healing response of HCECs on donor tissues in presence of EVs. *p < 0.05.

HCEC-12 with EVs slowed down the wound healing response (Figure 6A) and, it was found to be statistically significantly different at day 1 (Figure 6B). Wound was completely healed within 3 days in both the groups (Table 2).

**Table 2:** Percentage wound healing *in vitro* and on *ex vivo* human, pig and rabbit tissues.

**Wound healing (%)**	**HCEC-12 cells without EVs**	**HCEC-12 cells with EVs**
Day 1	70 ± 2	80 ± 2
Day 2	15 ± 1	20 ± 1
Day 3	0 ± 0	0 ± 0
**Wound healing (%)**	**Human tissues without EVs**	**Human tissues with EVs**
Day 1	88 ± 1	90 ± 2
Day 2	57 ± 3	65 ± 3
Day 3	35 ± 1	47 ± 6
Day 4	3 ± 1	13 ± 2
Day 5	0 ± 0	0 ± 0
**Wound healing (%)**	**Pig tissue without human EVs**	**Pig tissue with human EVs**
Day 1	77 ± 5	92 ± 4
Day 2	33 ± 5	78 ± 6
Day 3	2 ± 2	26 ± 1
Day 4	0 ± 0	0 ± 0
**Wound healing (%)**	**Rabbit tissue without human EVs**	**Rabbit tissue with human EVs**
Day 1	81 ± 17	77 ± 12
Day 2	3 ± 2	18 ± 4
Day 3	0 ± 0	3 ± 1

#### Wound Healing Rate on *ex vivo* Human Donor Corneas (n = 6)

Human donor corneas without EVs showed faster wound healing response compared with the cells with EVs (Figure 6C). A statistical significance was observed at day 3 and 4 where the cells without EVs showed faster wound healing compared to the cells with EVs (Figure 6D) (Table 2).

#### Wound Healing Response on *ex vivo* Porcine and Rabbit Corneas With and Without HCEC-12 Derived EVs (n = 6 Each)

**Figure 7:**
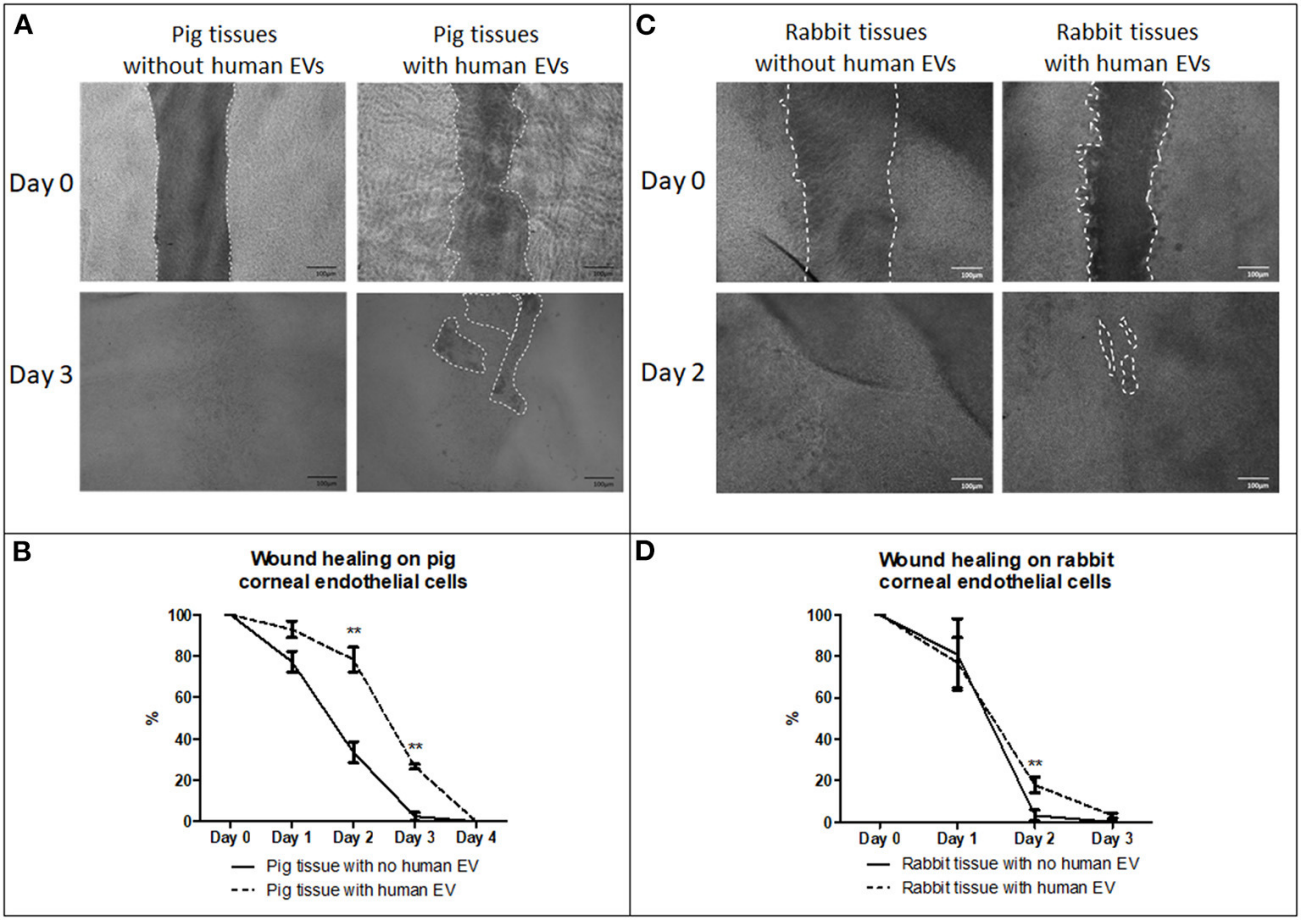
Effect of EVs derived from HCEC-12 cells on *ex vivo* porcine and rabbit corneas. **(A)** slow wound healing response was observed on porcine corneal endothelial cells when exposed to HCEC-12 derived EVs, **(B)** which showed statistical significance. Similar trend was observed on, **(C)** the rabbit tissues when exposed to EVs with **(D)** significantly slow wound healing response when exposed to HCEC-12 derived EVs. *p < 0.05; **p < 0.01.

Porcine corneas with human EVs showed a slow wound healing response (Figure 7A) that was statistically significant at days 2 and 3 (Figure 7B). Rabbit corneas with human EVs slowed the wound healing response (Figure 7C) and was found to be significantly different at day 2 (Figure 7D) (Table 2). This indicated that human EVs affect the migration of endothelial cells of other species that have otherwise shown to possess natural capacity to proliferate *in vivo*.

### Next Generation Sequencing of EVs Derived From HCEC-12 Cells

**Figure 8:**
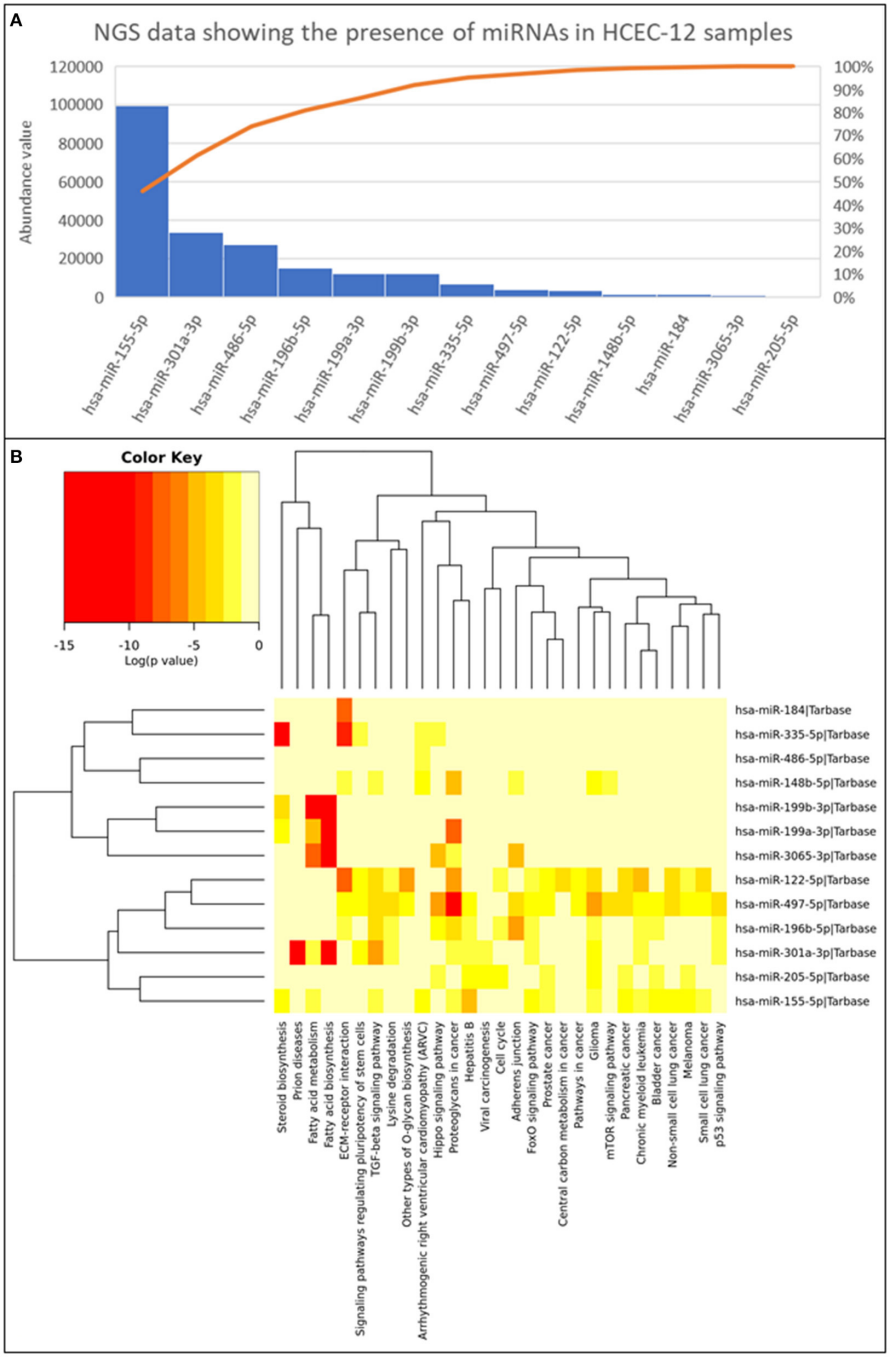
Next generation sequencing data showing the presence of 13 miRNAs and their abundance values derived from HCEC-12 cells. **(A)** The Pareto chart plot shows the distribution of the data in descending order of frequency with a cumulative line on a secondary axis as a percentage of the total. **(B)** Heatmap of all the miRNAs and their associated pathways.

Next generation sequencing showed 13 microRNAs (Figure 8A) from HCEC-12 derived EVs. It was observed that some of these microRNAs were actively involved in cell cycle pathway and inducing cellular apoptosis (Figure 8B).

## Discussion

Exosomes derived from different cell types has been involved in a wide panoply of therapeutic functions (13). Exosomes are enriched in major histocompatibility complexes and do not respond to immunosuppressive molecules thus encouraging their use as therapeutic agents (42–45). This is mainly due to their critical role in the transfer of bioactive molecules within and between tissues. However, the challenge remains in learning the effect of EVs derived from corneal endothelial cells. Anatomically, the posterior cornea is placed in an enclosed environment compared to the ocular surface. EVs therefore have a higher chance of retention in the anterior chamber than on the surface as they get washed by continuous tear flow. Therefore, the release and the effects of the EVs derived from corneal endothelial cells remain an interesting area of investigation.

In our study, the characterization i.e., size distribution and, positive expression of CD63 and TSG101 and, negative expression of GRP94 indicated that the isolated sample had a heterogeneous mixture of EVs, including exosomes. However, purifying and enriching exosomes will be crucial if intended for re-modeling the exosomal cargo for therapeutic purpose. Dil labeling showed that the cells take up small number of EVs at 3, 6, and 12 h however, a higher cellular uptake of EVs was observed at 24 and 48 h. EVs require longer time to be completely internalized in the cells (Supplementary Figure 1C) however, the localization appears to be dispersed, as the EVs were observed both, on the surface, in the cytoplasm and on the nucleus (Supplementary Figures 1A,B). The EVs did not show any change in cell proliferation after 48 h (unpublished data) therefore, to observe the chronic effect, the EVs were added every alternate day while refreshing the media. This showed that the EVs have a shorter life span to deliver the cargo inside the cell and continuous addition of EVs is required to see a long-lasting detrimental effect. This would be extremely important for the development of EV therapy. Continuous presence and uptake of EVs in the cell inhibits proliferation, cell doubling time and rate, further highlighting that the continuous release and uptake of EVs may result in reduced proliferative capacity of CECs *in vivo*. EVs at 10% concentration did not induce mortality to the normal functioning cell however, 100% concentration of EVs showed mortality of cells during the dose response study (data not shown). In addition, the expression of tight junction or pump function proteins are not significantly affected although the expression was not consistent throughout the surface. However, in an attempt to survive and function, the cells enlarged to cover the vacant space available due to loss of surrounding cells thus increasing polymorphism and pleomorphism. These features are like FECD cells i.e., the cells show polymegathism, further indicative of increased exosomal activity under stressed environment. Although EVs are released by the cells routinely, stress of any form could release excessive EVs from the CECs in the anterior chamber of the eye. This continuous release of EVs could result into uptake of more EVs by the cells resulting into overexpression of certain miRNAs that could possibly inhibit the proliferation of cells.

FECD which is one of the leading causes of corneal blindness and transplant has shown to be susceptible to oxidative DNA damage and oxidative stress-induced apoptosis than normal corneal endothelial cells. Increased activation of p53 in FECD has suggested that it mediates cell death in susceptible corneal endothelial cells. This means that p53 plays a critical role in complex mechanisms regulating oxidative-stress-induced apoptosis in FECD (46). Studies have also reported that excessive apoptosis may be an important mechanism in the pathogenesis of FECD (47). The cargo analysis in our study using NGS showed that hsa-miR-196b-5p is involved in p53 pathway. In addition, it has been observed that EVs influence immune activation through cell-to-cell communication, while oxidative stress enhances exosome release from stressed cells. In our study, we starved the cells to obtain a higher quantity of EVs for experiments with serum depleted media to enhance the release of EVs. This means that the cells, when stressed, release excess EVs (48–50). Our hypothesis here is that following oxidative stress, normal corneal cells start releasing excessive EVs, which contain factors that promote apoptosis. In addition, as endothelial cells are in a closed environment, continuous release of EVs and cellular uptake of excessive EVs may result in FECD. Therefore, the next challenge will be to investigate the specifics of each miRNA and reverse the inhibiting factor to increase the proliferation rate of these cells and to understand the pathogenesis of EV derived FECD.

CECs have shown specific properties with regards to wound healing. Primarily, the endothelium heals by cell migration followed by increased cell spreading. This process may be followed by endothelial-mesenchymal transformation. Cell proliferation, however, plays a secondary role (51). This could be a reason of the larger cell area induced by EVs. Moreover, the cells reached confluence with lower cell count which again highlights that the cells expanded in size but not in numbers. In terms of migration and wound healing response, cells supplemented with EVs showed reduced cell migration or wound closure compared to the cells without EVs, *in vitro*. These results were translated to *ex vivo* human corneas as well. It has been shown that porcine and rabbit corneal endothelial cells have a greater proliferative capacity than humans (23, 24, 26, 52). This baseline difference leads to earlier wound closure of these species compared to the humans. Both, porcine and rabbit endothelial cells treated with human EVs slowed the wound healing response. This further indicated that there are factors in the exosomal cargo of HCEC-12 derived EVs that reduce the proliferative and migration capability of the known proliferative cells of different species. Slow wound healing could be a result of stress induced by human EVs changing the microenvironment of the CECs in animal tissues and not necessarily the exosomal cargo itself *per se*. However, the EV crosstalk mechanism between human and porcine/rabbit needs to be further investigated.

EVs transfer the cargo from the originating cell to the receiving cell and influence various biological processes such as differentiation, migration, proliferation etc. The cargo, that includes DNA, RNA, mRNA, miRNA, proteins, and lipids, can modulate the cellular fate (53). miRNA have been found to be in abundance and responsible for the functions of multiple EV populations. It has been demonstrated that miRNAs act toward biological characteristics including proliferation, cellular apoptosis, migration, and tumorigenesis. In our study, we reported 13 miRNAs with their abundance values (Figure 8). Some of which were found to have key roles in cell cycle (hsa-miR-205-5p; hsa-miR-196b-5p; hsa-miR-122-5p), adherence junction (hsa-miR-196b-5p; hsa-miR-497-5p; hsa-miR-3065-3p; hsa-miR-148b-5p) and p53 signaling pathway (hsa-miR-301a-3p; hsa-miR-196b-5p; hsa-miR-497-5p). hsa-miR-196b-5p was found in all three biological functions however, its role is mainly known in the progression of cancer cells (54–57). It has not been studied in the ocular research thus needing extensive research.

The therapeutic efficacy of corneal endothelial cell derived EVs have not been studied and their secretions to restore tissue homeostasis enhancing tissue recovery, reparation, and regeneration needs attention. While many functions of EVs have been identified, investigations about EV functions in many specialized tissues of the eye are just at the preliminary stage. However, as we observed that the EVs inhibit the growth of HCEnCs, as an alternative, reprogramming the EVs to induce growth and proliferation of cells could be a potential future therapeutic approach. Enriching exosomes from EVs and utilizing them as a carrier for a desired therapeutic molecule/miRNA would further supplement the future development for the corneal endothelial treatment.

## Data Availability Statement

The datasets presented in this article are not readily available because they are in the internal server of UCL Institute of Ophthalmology. Requests to access the datasets should be directed to Sajjad Ahmad.

## Ethics Statement

Ethical review and approval was not required for the animal study because the study was performed on tissues from dead animals.

## Author Contributions

All authors listed have made a substantial, direct, and intellectual contribution to the work and approved it for publication.

## Funding

This study was fully funded by Moorfields Eye Charity grant (R170041A) to SA (Nov 2016-Oct 2021). The publication and open access of this article has been enabled by a grant from Moorfields Eye Charity [GR001457].

## Conflict of Interest

The authors declare that the research was conducted in the absence of any commercial or financial relationships that could be construed as a potential conflict of interest.

## Publisher's Note

All claims expressed in this article are solely those of the authors and do not necessarily represent those of their affiliated organizations, or those of the publisher, the editors and the reviewers. Any product that may be evaluated in this article, or claim that may be made by its manufacturer, is not guaranteed or endorsed by the publisher.

## Supplementary Material

Supplementary Figure 1 Cellular uptake and 3D imaging using confocal microscopy. **(A)** A z-stack 3D image showing internalization / localization of EVs in the cell. **(B)** Cellular uptake of Dil labeled EVs (stained in red) at different time points. **(C)** Imagestream analysis showing the uptake and internalization of Dil labeled EVs at different time points.

Supplementary Video 1 Nanosight analysis showing the presence of EVs derived from HCEC-12 cells.

## References

[1] Parekh M Romano V Hassanin K Testa V Wongvisavavit R Ferrari S. Biomaterials for corneal endothelial cell culture and tissue engineering. J Tissue Eng. (2021) 12:2041731421990536. 10.1177/2041731421990536 33643603 PMC7894589

[2] Bourne WM. Biology of the corneal endothelium in health and disease. Eye. (2003) 17:912–8. 10.1038/sj.eye.6700559 14631396

[3] Stiemke MM Edelhauser HF Geroski DH. The developing corneal endothelium: correlation of morphology, hydration and Na/K ATPase pump site density. Curr Eye Res. (2009) 10:145–56. 10.3109/02713689109001742 1645240

[4] Senoo T Joyce NC. Cell cycle kinetics in corneal endothelium from old and young donors. Invest Ophthalmol Vis Sci. (2000) 41:660–7. 10711678

[5] Joyce NC Navon SE Roy S Zieske JD. Expression of cell cycle-associated proteins in human and rabbit corneal endothelium in situ. Invest Ophthalmol Vis Sci. (1996) 37:1566–75. 8675399

[6] Bahn CF Glassman RM MacCallum DK Lillie JH Meyer RF Robinson BJ. Postnatal development of corneal endothelium. Invest Ophthalmol Vis Sci. (1986) 27:44–51. 3941037

[7] Nucci P Brancato R Mets MB Shevell SK. Normal endothelial cell density range in childhood. Arch Ophthalmol. (1990) 108:247–8. 10.1001/archopht.1990.01070040099039 2302110

[8] Yee RW Matsuda M Schultz RO Edelhauser HF. Changes in the normal corneal endothelial cellular pattern as a function of age. Curr Eye Res. (2009) 4:671–8. 10.3109/02713688509017661 4028790

[9] Wilson SE Bourne WM Brubaker RF. Effect of dexamethasone on corneal endothelial function in Fuchs' dystrophy. Invest Ophthalmol Vis Sci. (1988) 29:357–61. 3257749

[10] Krachmer JH. Posterior polymorphous corneal dystrophy: a disease characterized by epithelial-like endothelial cells which influence management and prognosis. Trans Am Ophthalmol Soc. (1985) 83:413–75. 3914130 PMC1298709

[11] Pandrowala H Bansal A Vemuganti GK Rao GN. Frequency, distribution, and outcome of keratoplasty for corneal dystrophies at a tertiary eye care center in South India. Cornea. (2004) 23:541–6. 10.1097/01.ico.0000126324.58884.b9 15256989

[12] Levy SG McCartney ACE Baghai MH Barrett MC Moss J. Pathology of the iridocorneal–endothelial syndrome. the ICE-cell. Invest Ophthalmol Vis Sci. (1995) 36:2592–601. 7499082

[13] Nuzzi R Buono L Scalabrin S DeIuliis M Bussolati B. Effect of stem cell-derived extracellular vesicles on damaged human corneal endothelial cells. Stem Cells Int. (2021) 2021:6644463. 10.1155/2021/6644463 33531909 PMC7834816

[14] Miyamoto T Sumioka T Saika S. Endothelial mesenchymal transition: a therapeutic target in retrocorneal membrane. Cornea. (2010) 29:S52–6. 10.1097/ICO.0b013e3181efe36a 20935543

[15] Polse KS Brand RJ Cohen SR Guillon M. Hypoxic effects on corneal morphology and function. Invest Ophthalmol Vis Sci. (1990) 31:1542–54. 2387685

[16] Gain P Jullienne R He Z Aldossary M Acquart S Cognasse F. Global survey of corneal transplantation and eye banking. JAMA Ophthalmol. (2016) 134:167–73. 10.1001/jamaophthalmol.2015.4776 26633035

[17] Okumura N Koizumi N Ueno M Sakamoto Y Takahashi H Tsuchiya H. ROCK inhibitor converts corneal endothelial cells into a phenotype capable of regenerating in vivo endothelial tissue. Am J Pathol. (2012) 181:268–77. 10.1016/j.ajpath.2012.03.033 22704232

[18] Peh GSL Ang HP Lwin CN Adnan K George BL Seah XY. Regulatory compliant tissue-engineered human corneal endothelial grafts restore corneal function of rabbits with bullous keratopathy. Sci Rep. (2017) 7:14149. 10.1038/s41598-017-14723-z 29074873 PMC5658403

[19] Kinoshita S Koizumi N Ueno M Okumura N Imai K Tanaka H. Injection of cultured cells with a ROCK inhibitor for bullous keratopathy. N Engl J Med. (2018) 378:995–1003. 10.1056/NEJMoa1712770 29539291

[20] Parekh M Peh GSL Mehta JS Ahmad S Ponzin D Ferrari S. Effects of corneal preservation conditions on human corneal endothelial cell culture. Exp Eye Res. (2019) 179:93–101. 10.1016/j.exer.2018.11.007 30414971

[21] Van Horn DL Hyndicek RA. Endothelial wound repair in primate cornea. Exp Eye Res. (1975) 21:113–24. 10.1016/0014-4835(75)90076-7 809290

[22] Khodadoust AA Green K. Physiological function of regenerating endothelium. Invest Ophthalmol Vis Sci. (1976) 15:96–101. 1245388

[23] Van Horn DL Sendele DD Seidemen S Buco PJ. Regenerative capacity of the corneal endothelium in rabbit and cat. Invest Ophthalmol Visual Sci. (1977) 17:597–613. 873721

[24] Gospodarowicz D Greenburg G Alvarado J. Transplantation of cultured bovine corneal endothelial cells to rabbit cornea: clinical implications for human studies. Proc Natl Acad Sci U S A. (1979) 76:464–8. 10.1073/pnas.76.1.464 370830 PMC382961

[25] Marquez-Curtis LA McGann LE Elliott JAW. Expansion and cryopreservation of porcine and human corneal endothelial cells. Cryobiology. (2017) 77:1–13. 10.1016/j.cryobiol.2017.04.012 28465186

[26] Nicholls S Bailey M Mitchard L Dick AD. Can the corneal endothelium of the pig proliferate in vivo? ACTA Ophthal. (2009). 10.1111/j.1755-3768.2009.2271.x22467584

[27] Grange C Tritta S Tapparo M Cedrino M Tetta C Camussi G. Stem cell-derived extracellular vesicles inhibit and revert fibrosis progression in a mouse model of diabetic nephropathy. Sci Rep. (2019) 9:1–13. 10.1038/s41598-019-41100-9 30872726 PMC6418239

[28] Giebel B Kordelas L Börger V. Clinical potential of mesenchymal stem/stromal cell-derived extracellular vesicles. Stem Cell Investigation. (2017) 4:84. 10.21037/sci.2017.09.06 29167805 PMC5676188

[29] Kalluri R LeBleu VS. The biology, function, and biomedical applications of exosomes. Science. (2020) 367:eaau6977. 10.1126/science.aau6977 32029601 PMC7717626

[30] Parekh M Peh G Mehta JS Ramos T Ponzin D Ahmad S. Passaging capability of human corneal endothelial cells derived from old donors with and without accelerating cell attachment. Exp Eye Res. (2019) 189:107814. 10.1016/j.exer.2019.107814 31560924

[31] Parekh M Graceffa V Bertolin M Elbadawy H Salvalaio G Ruzza A. Reconstruction and regeneration of corneal endothelium: a review on current methods and future aspects. J Cell Sci Ther. (2013) 4:146. 10.4172/2157-7013.1000146

[32] Parekh M Ferrari S Sheridan C Kaye S Ahmad S. Concise review: an update on the culture of human corneal endothelial cells for transplantation. Stem Cells Transl Med. (2016) 5:258–64. 10.5966/sctm.2015-0181 26702128 PMC4729556

[33] Parekh M Romano V Ruzza A Kaye SB Ponzin D Ahmad S. Culturing discarded peripheral human corneal endothelial cells from the tissues deemed for preloaded DMEK transplants. Cornea. (2019) 38:1175–81. 10.1097/ICO.0000000000001998 31169610

[34] Peh GS Beuerman RW Colman A Tan D Mehta JS. Human corneal endothelial cell expansion for corneal endothelium transplantation: an overview. Transplantation. (2011) 91:811–9. 10.1097/TP.0b013e3182111f01 21358368

[35] Peh GSL Chng Z Ang HP Cheng TYD Adnan K Seah XY. Propagation of human corneal endothelial cells: a novel dual media approach. Cell Transplant. (2013) 24:287–304. 10.3727/096368913X675719 24268186

[36] Parekh M Ruzza A Ferrari S Ponzin D. Preservation of preloaded DMEK lenticules in dextran and non-dextran-based organ culture medium. J Ophthalmol. (2016) 2016:5830835. 10.1155/2016/5830835 27994884 PMC5138458

[37] Parekh M Ramos T O'Sullivan F Meleady P Ferrari S Ponzin D. Human corneal endothelial cells from older donors can be cultured and passaged on cell-derived extracellular matrix. Acta Ophthalmol. (2021) 99:e512–22. 10.1111/aos.14614 32914525

[38] Livak KJ Schmittgen TD. Analysis of relative gene expression data using real-time quantitative PCR and the 2(–Delta Delta C(T)) method. Methods. (2011) 25:402–8. 10.1006/meth.2001.1262 11846609

[39] Kuhn RM Haussler D Kent WJ. The UCSC genome browser and associated tools. Brief Bioinform. (2013) 14:144–61. 10.1093/bib/bbs038 22908213 PMC3603215

[40] Langmead B Salzberg SL. Fast gapped-read alignment with Bowtie 2. Nat Methods. (2012) 9:357–9. 10.1038/nmeth.1923 22388286 PMC3322381

[41] Li H Handsaker B Wysoker A Fennell T Ruan J Homer N. The sequence alignment/map format and SAMtools. Bioinformatics. (2009) 25:2078–9. 10.1093/bioinformatics/btp352 19505943 PMC2723002

[42] Escudier B Dorval T Chaput N Andre F Caby MP Novault S. Vaccination of metastatic melanoma patients with autologous dendritic cell (DC) derived-exosomes: results of thefirst phase I clinical trial. J Transl Med. (2005) 3:10. 10.1186/1479-5876-3-10 15740633 PMC554765

[43] Ratajczak J Meikus K Kucia M Zhang J Reca R Dvorak P. Embryonic stem cell-derived microvesicles reprogram hematopoietic progenitors: evidence for horizontal transfer of mRNA and protein delivery. Leukemia. (2006) 20:847. 10.1038/sj.leu.2404132 16453000

[44] Ratajczak J Wysoczynski M Hayek F Janowska-Wieczorek A Ratajczak MZ. Membrane-derived microvesicles: important and underappreciated mediators of cell-to-cell communication. Leukemia. (2006) 20:1487–95. 10.1038/sj.leu.2404296 16791265

[45] Katsman D Stackpole EJ Domin DR Farber DB. Embryonic stem cell-derived microvesicles induce gene expression changes in Muller cells of the retina. PLoS ONE. (2012) 7:e50417. 10.1371/journal.pone.0050417 23226281 PMC3511553

[46] Azizi B Fuchsluger T Schmedt T Chen Y Jurkunas U. p53-regulated increase in oxidative-stress–induced apoptosis in fuchs endothelial corneal dystrophy: a native tissue model. Invest Ophthalmol Vis Sci. (2011) 52:9291–7. 10.1167/iovs.11-8312 22064994 PMC3250096

[47] Li QJ Ashraf MF Shen DF Green WR Stark WJ Chan CC. The role of apoptosis in the pathogenesis of fuchs endothelial dystrophy of the cornea. Arch Ophthalmol. (2001) 119:1597–604. 10.1001/archopht.119.11.1597 11709009

[48] Eldh M Ekström K Valadi H Sjöstrand M Olsson B Jernås M. Exosomes communicate protective messages during oxidative stress; possible role of exosomal shuttle RNA. PLoS ONE. (2010) 5:e15353. 10.1371/journal.pone.0015353 21179422 PMC3003701

[49] Hedlund M Nagaeva O Kargl D Baranov V Nilsson ML. Thermal- and oxidative stress causes enhanced release of NKG2D ligand-bearing immunosuppressive exosomes in leukemia/lymphoma T and B cells. PLoS ONE. (2011) 6:e16899. 10.1371/journal.pone.0016899 21364924 PMC3045385

[50] Chettimada S Lorenz DR Misra V Dillon ST Reeves RK Manickam C. Exosome markers associated with immune activation and oxidative stress in HIV patients on antiretroviral therapy. Sci Rep. (2018) 8:7227. 10.1038/s41598-018-25515-4 29740045 PMC5940833

[51] Lubinov AV Saghizadeh M. Progress in corneal wound healing. Prog Retin Eye Res. (2015) 49:17–45. 10.1016/j.preteyeres.2015.07.002 26197361 PMC4651844

[52] Smeringaiova I Merjava SR Stranak Z Studeny P Bednar J Jirsova K. Endothelial wound repair of the organ-cultured porcine corneas. Curr Eye Res. (2018) 43:856–65. 10.1080/02713683.2018.1458883 29648937

[53] Valadi H Ekstrom K Bossios A Sjostrand M Lee JJ Lotvall JO. Exosome-mediated transfer of mRNAs and microRNAs is a novel mechanism of genetic exchange between cells. Nat Cell Biol. (2007) 9:654–9. 10.1038/ncb1596 17486113

[54] Xin H Wang C Chi Y Liu Z. MicroRNA-196b-5p promotes malignant progression of colorectal cancer by targeting ING5. Cancer Cell Int. (2020) 20:119. 10.1186/s12935-020-01200-3 32308564 PMC7149860

[55] Zhang L Luo B Dang YW He RQ Peng ZG Chen Z. Clinical significance of microRNA-196b-5p in hepatocellular carcinoma and its potential molecular mechanism. J Cancer. (2019) 10:5355–70. 10.7150/jca.29293 31632480 PMC6775707

[56] Lee SW Park KC Kim JG Moon SJ Kang SB Lee DS. Dysregulation of MicroRNA-196b-5p and MicroRNA-375 in gastric cancer. J Gastric Cancer. (2016) 16:221–9. 10.5230/jgc.2016.16.4.221 28053808 PMC5206312

[57] Li J Wang L He F Li B Han R. Long noncoding RNA LINC00629 restrains the progression of gastric cancer by upregulating AQP4 through competitively binding to miR-196b-5p. J Cell Physiol. (2020) 235:2973–85. 10.1002/jcp.29203 31674022

