# Supplementary material for: Extracellular Vesicles Derived From Human Corneal Endothelial Cells Inhibit Proliferation of Human Corneal Endothelial Cells
Source: Front Med (Lausanne). 2022 Feb 4;8:753555. doi: 10.3389/fmed.2021.753555 (PMC8854366; doi:10.3389/fmed.2021.753555)
Supplement: Supplementary file 1 [file Table_1.docx]

| Antibody | Clone | Host | Isotype | Manufacturer | Dilution |
| --- | --- | --- | --- | --- | --- |
| CD63 | Polyclonal | Mouse | IgG | Santa Cruz Biotechnology | 1:1000 |
| TSG101 | 4A10 | Mouse | IgG1 | Abcam | 1:1000 |
| GRP94 | 9G10 | Rat | IgG2a | Abcam | 1:1000 |
| IgG | --- | Mouse | --- | ThermoFisher Scientific | 0.2µg/µL |
| IgG1 | --- | Mouse | --- | ThermoFisher Scientific | 1µg/µL |
| IgG2 | --- | Rat | --- | ThermoFisher Scientific | 1µg/µL |

Supplementary table 1 – List of primary antibodies used for exosome characterization using flow cytometry.

| Antibody | Clone | Host | Reactivity | Conjugate | Manufacturer | Dilution |
| --- | --- | --- | --- | --- | --- | --- |
| Anti-mouse | A-11005 | Goat | Mouse | FITC | Life Technologies | 1:1000 |
| Anti-rat | F7512 | Sheep | Rabbit | FITC | Sigma-Aldrich | 1:1000 |

Supplementary table 2 - List of secondary antibodies used for exosome characterization using flow cytometry.
