# Supplementary figures and images for: Extracellular Vesicles Derived From Human Corneal Endothelial Cells Inhibit Proliferation of Human Corneal Endothelial Cells
Source: Front Med (Lausanne). 2022 Feb 4;8:753555. doi: 10.3389/fmed.2021.753555 (PMC8854366; doi:10.3389/fmed.2021.753555)

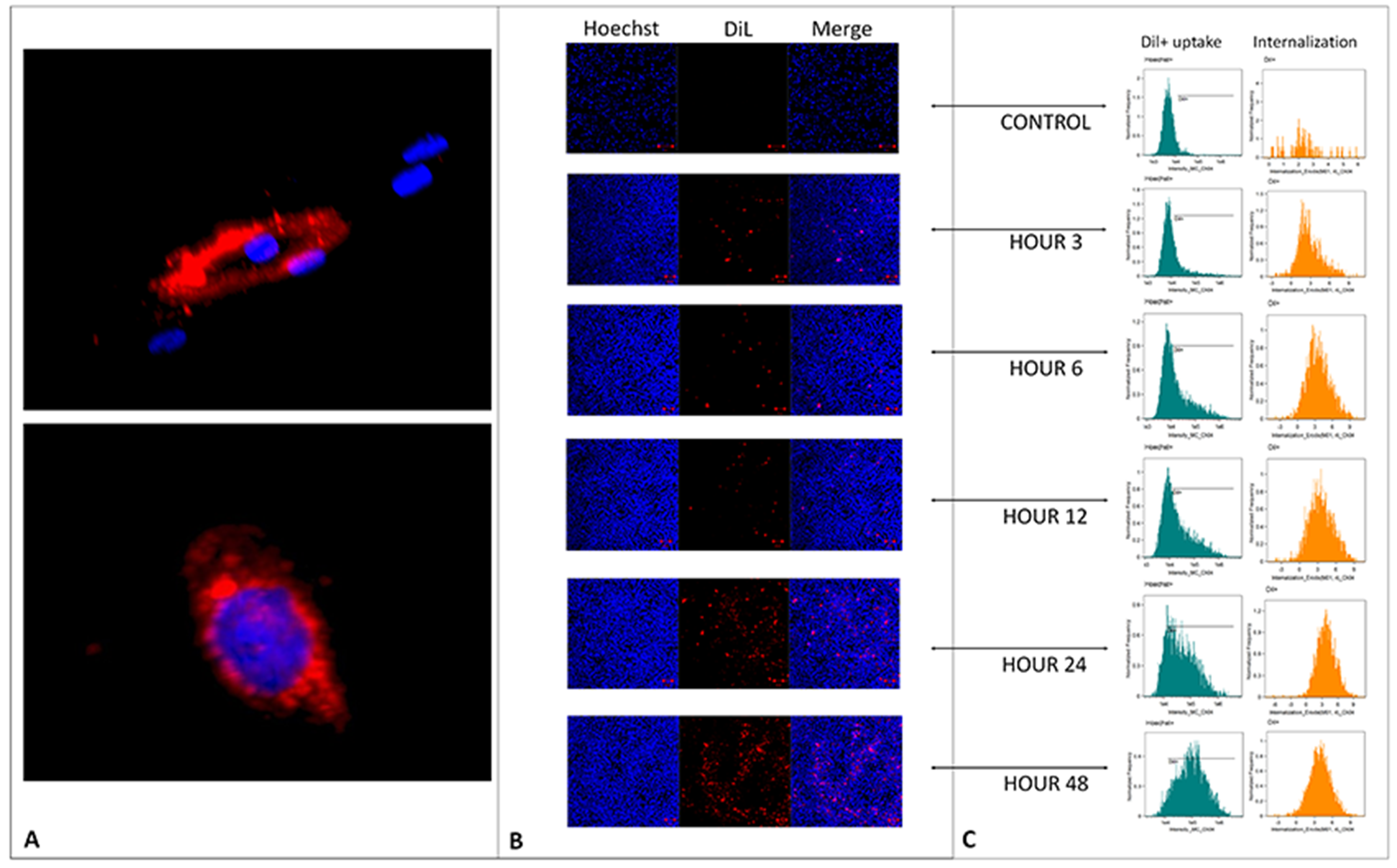

Supplement: Supplementary Figure 1 — Cellular uptake and 3D imaging using confocal microscopy. (A) A z-stack 3D image showing internalization / localization of EVs in the cell. (B) Cellular uptake of Dil labeled EVs (stained in red) at different time points. (C) Imagestream analysis showing the uptake and internalization of Dil labeled EVs at different time points. [file Image_1.TIF]
